# Transcellular biosynthesis of leukotriene B_4_ orchestrates neutrophil swarming to fungi

**DOI:** 10.1016/j.isci.2022.105226

**Published:** 2022-09-28

**Authors:** Alex Hopke, Tian Lin, Allison K. Scherer, Ashley E. Shay, Kyle D. Timmer, Brittany Wilson-Mifsud, Michael K. Mansour, Charles N. Serhan, Daniel Irimia, Bryan P. Hurley

**Affiliations:** 1 Center for Engineering in Medicine and Surgery, Massachusetts General Hospital, Boston, MA 02129, USA; 2 Harvard Medical School, Boston, MA 02115, USA; 3 Shriners Hospital for Children, Boston, MA 02114, USA; 4 Mucosal Immunology and Biology Research Center, Massachusetts General Hospital, Boston, MA 02114, USA; 5 Division of Infectious Diseases, Massachusetts General Hospital, Boston, MA 02114, USA; 6 Center for Experimental Therapeutics and Reperfusion Injury, Department of Anesthesiology, Perioperative, and Pain Medicine, Brigham and Women’s Hospital and Harvard Medical School, Boston, MA 02115, USA

**Keywords:** Immunology, Cell biology

## Abstract

Neutrophil swarming is an emergent host defense mechanism triggered by targets larger than a single neutrophil’s capacity to phagocytose. Swarming synergizes neutrophil functions, including chemotaxis, phagocytosis, and reactive oxygen species (ROS) production, and coordinates their deployment by many interacting neutrophils. The potent inflammatory lipid mediator leukotriene B_4_ (LTB_4_) has been established as central to orchestrating neutrophil activities during swarming. However, the details regarding how this eicosanoid choreographs the neutrophils involved in swarming are not well explained. Here we leverage microfluidics, genetically deficient mouse cells, and targeted metabolipidomic analysis to demonstrate that transcellular biosynthesis occurs among neutrophils to generate LTB_4_. Furthermore, transcellular biosynthesis is an entirely sufficient means of generating LTB_4_ for the purposes of orchestrating neutrophil swarming. These results further our understanding of how neutrophils coordinate their activities during swarming, which will be critical in the design of eventual therapies that can harness the power of swarming behavior.

## Highlights

• Mouse neutrophils unable to biosynthesize LTB_4_ fail to swarm or contain fungi
• Exogenous LTB_4_ restores phagocytosis and ROS production but not swarming
• Transcellular LTB_4_ biosynthesis restores swarming
• Transcellular LTB_4_ biosynthesis controls fungal growth

## Graphical abstract

**Figure:**
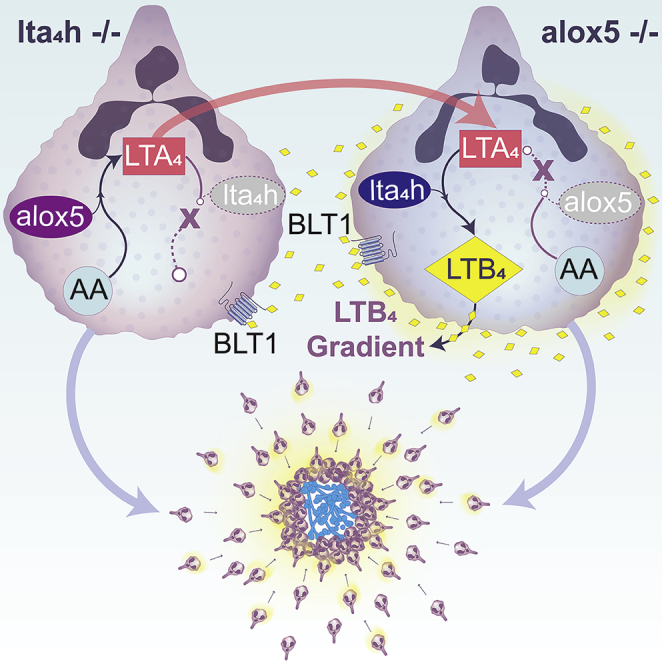

## Introduction

Neutrophils have long been known for their critical role in the protection against fungal infections, featuring an armament of antimicrobial defenses (Desai and Lionakis, 2018). Among the most recently described is the behavior of swarming, during which neutrophils coordinate their own exponential recruitment to concentrate antimicrobial action against large targets (Kienle and Lämmermann, 2016). The role of LTB_4_ as a critical mediator of neutrophil swarming has been well established in mice and humans by lipidomic analysis, antagonizing LTB_4_ receptors, inhibiting LTB_4_ biosynthesis, genetically manipulating intracellular LTB_4_ signaling, and disrupting LTB_4_ biosynthesis pathways (Hopke et al., 2020; Lammermann et al., 2013; Malawista et al., 2008; Reategui et al., 2017). Despite these advances, details of LTB_4_ biosynthesis and transport by neutrophils while swarming remain largely unexplored. A hint to potential complexity is provided by earlier studies showing that during inflammation, neutrophils exchange significant quantities of eicosanoid intermediates with other immune and non-immune cells (Serhan et al., 1984a, 2020). Transcellular eicosanoid biosynthesis adds flexibility and robustness to coordinate responses resulting from interactions between neutrophils and platelets (Hopke et al., 2020; Kienle et al., 2021; Lammermann et al., 2013; Reategui et al., 2017), neutrophils and red blood cells (Stern and Serhan, 1989), neutrophils and endothelial cells (Claesson and Haeggstrom, 1988), neutrophils and airway epithelial cells, (Bigby et al., 1989) neutrophils and epidermal cells (Sola et al., 1992), neutrophils and lymphocytes (Odlander et al., 1988), etc. However, in this rich context, it is unknown if transcellular LTB_4_ biosynthesis plays a role in coordinating neutrophil-neutrophil interactions during swarming. Here, we employ microfluidics, genetically deficient mouse cells, and targeted metabolipidomic analysis to probe the role of transcellular biosynthesis of LTB_4_ during neutrophil swarming and restriction of pathogen growth.

## Results

**Figure 1:**
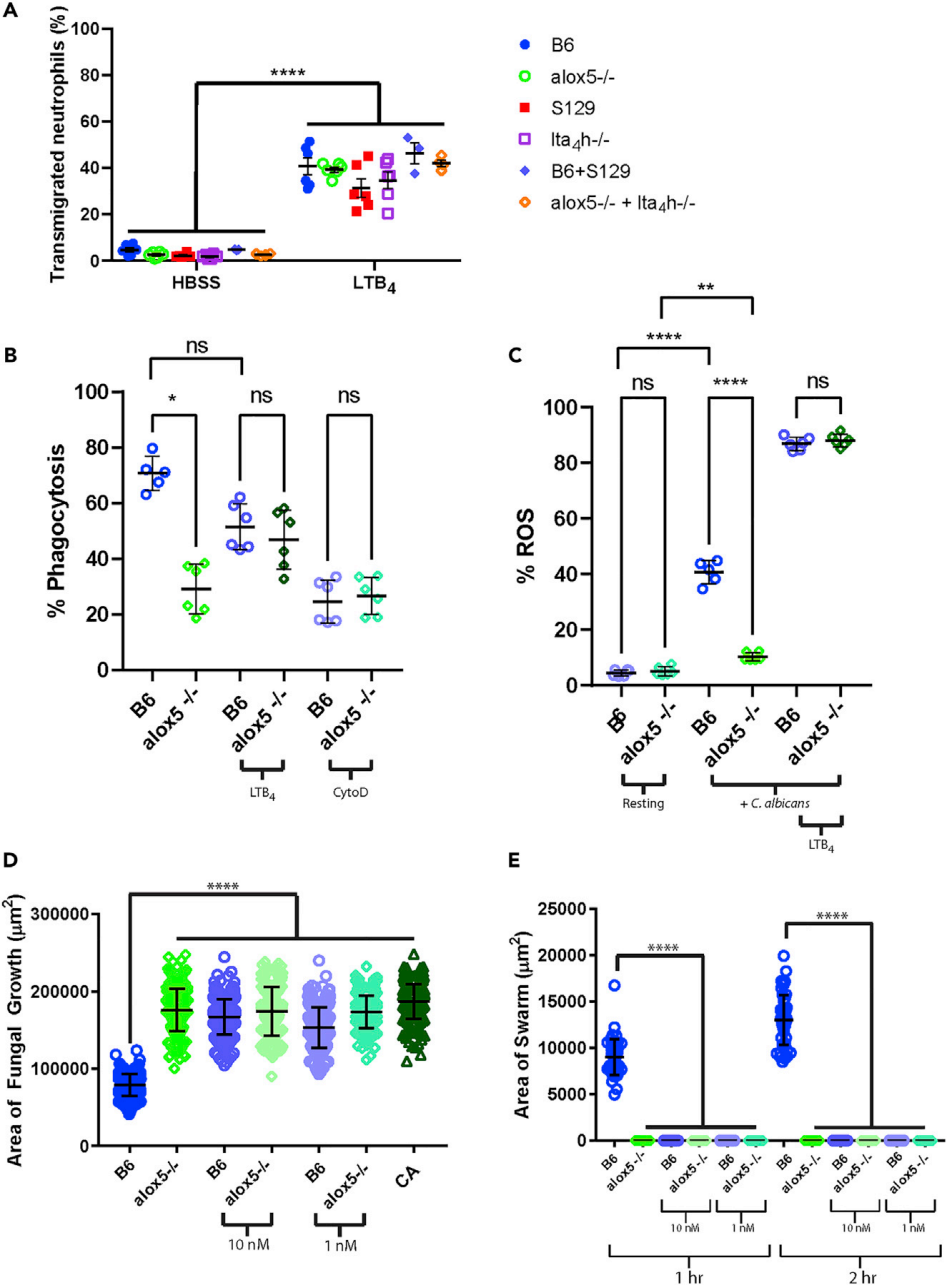
Neutrophil swarming is absent and cannot be restored by LTB_4_ in neutrophils from alox5^−/−^ and lta_4_h^−/−^ mice, whereas common neutrophil functions are comparable to wild-type or could be restored by LTB_4_ (A) Transmigration toward LTB_4_ (0.2 ng/mL) through a membrane with 3 μm pores is comparable for bone marrow cells from B6 (C57/BL6 mice, the wild-type control for the alox5^−/−^ mice) and alox5^−/−^ or S129 (129S1/SvImJ mice, the wildtype control for the lta4h−/− mice) and lta_4_h^−/−^ mice. There are no significant differences within the HBSS or the LTB_4_ groups. (B and C) The ability to phagocytose (B)*C. albicans* and produce ROS (C) by enriched neutrophils from alox5^−/−^ mice is restored by LTB_4_ (0.6 nM) to levels comparable to C57 mice. N = 6 mice per genotype across 2 independent experiments. Phagocytosis events were differentiated from cell surface adherence events with the cytoskeletal inhibitor cytochalasin D (CytoD) at a concentration of 30 μM. (D and E) Neutrophils enriched from the bone marrow of B6 and alox5^−/−^ mice, 500,000 neutrophils per genotype. Concentrations of 10 nM or 1 nM refer to LTB_4_. (D) The amount of fungal growth of *C. albicans* was quantified at 16 h after the start of the assay. CA refers to Candida alone, a condition in which only media is added to live *Candida albicans* targets. N≥ 282 swarms across three independent experiments. (E) The area covered by the neutrophil swarm was quantified at the indicated timepoint. N = 48 swarms across 3 independent experiments. Mean and SD are shown, except for A, which is SEM ∗∗∗∗p≤0.0001 by Kruskal-Wallis or one way ANOVA. See also Figure S1.

We tested the ability of LTB_4_ to restore the functions of neutrophils from mice with knockout genotypes at two critical steps in the LTB_4_ synthesis pathway: 5-lipoxygenase (5-LOX) and leukotriene A_4_ hydrolase (LTA_4_H), encoded by *alox5* and *lta4h,* respectively (Wan et al., 2017). We verified that there were no significant differences in expression of the primary LTB_4_ receptor BLT1 (Figure S1A) and no differences in chemotaxis toward LTB_4_ (Figure 1A) between knockout and wild-type cell counterparts (C57/BL6 vs. *alox5*^−/−^ and S129 vs. *lta4h*^−/−^). Consistent with the essential roles of LTB_4_ in stimulating neutrophil phagocytosis and ROS production, we found significant differences in phagocytosis and ROS production between knockout and wild-type cells. These differences were corrected by the addition of exogenous LTB_4_ (Figures 1B, 1C and S1B).

Next, we tested the swarming of mouse neutrophils triggered by 100 μm diameter clusters of live *Candida albicans*, a common example of an opportunistic fungal pathogen. We found swarming against these clusters as well as restriction of their growth to be completely defective in *alox5*^−/−^ cells, and these functions were not restored by the addition of LTB_4_ (Figures 1D and 1E). In addition, the application of exogenous LTB_4_ appeared to disrupt the ability of wild-type cells to swarm effectively and restrict fungal growth (Figures 1D and 1E). This result was surprising as it was established earlier that the process of swarming is LTB_4_-signaling dependent (Hopke et al., 2020; Kienle et al., 2021; Lammermann et al., 2013; Malawista et al., 2008; Reategui et al., 2017). Our results confirm a critical role for LTB_4_ signaling, as blocking of the primary LTB4 receptor BLT1 disrupts swarming and restriction of *C. albicans* growth (Figures S2A and S2B). Of interest, LTB_4_ levels appeared higher in the anti-BLT1 treated condition (Figure S2C). This may be due to an inability of the BLT1 receptor to bind and remove LTB_4_ from the media. Despite this increased LTB_4_, swarming is completely compromised, demonstrating the importance of sensing LTB_4_ to an effective swarm response. Together, these results highlight the unique requirements for LTB_4_ during swarming. These requirements depend not only on the presence of LTB_4_ as observed with chemotaxis, phagocytosis, and ROS production, but also on context.

**Figure 2:**
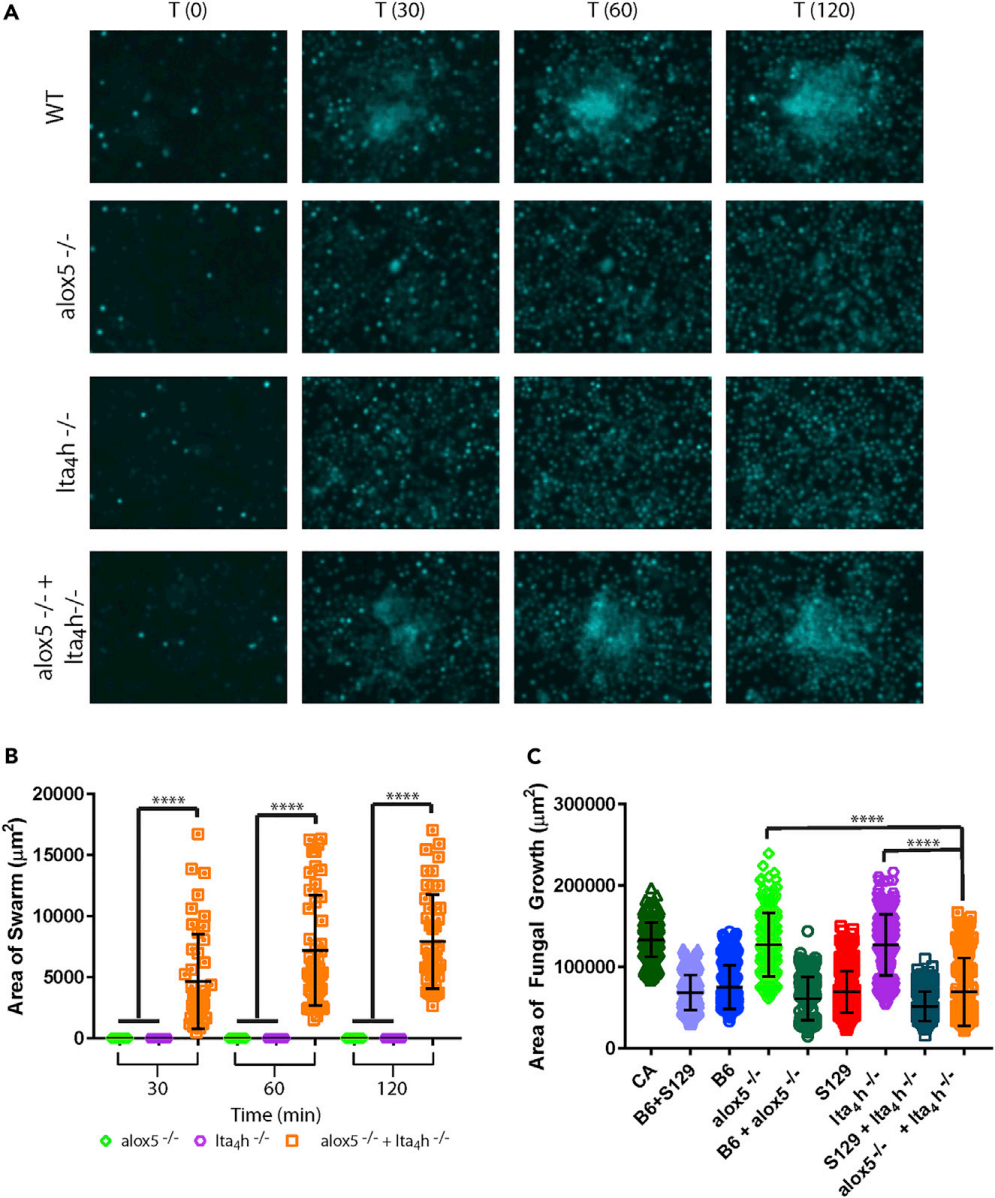
Swarming responses against *C. albicans* are absent in homogeneous and restored in heterogeneous populations of bone marrow cells (A) Fluorescence imaging (Hoechst) of mouse bone marrow cells swarming against live *C. albicans* target. 500,000 bone marrow cells from wild-type, alox5^−/−^, lta_4_h^−/−^, and equal numbers of alox5^−/−^ + lta_4_h^−/−^ mice were added to swarming arrays. Representative images of swarming from wild-type cells, alox5^−/−^ or lta_4_h^−/−^ cells alone and alox5^−/−^ + lta_4_h^−/−^ mixed together 1:1 are shown. T is in minutes. (B) The size of the neutrophil swarms formed against *C. albicans* targets was quantified over time. N = 48 swarms across 3 independent experiments. (C) The amount of fungal growth of *C. albicans* was quantified 16 h after the start of the assay. N≥254 targets across three independent experiments. Mean and SD are shown. ∗∗∗∗p≤0.0001 by Kruskal-Wallis post-test. See also Figures S2 and S3.

Further investigation of the relationship between LTB_4_ and neutrophil swarming revealed that mixing the bone marrow cells derived from *alox5*^−/−^ and *lta4h*^−/−^ mice in a 1:1 ratio restores their capacity to swarm (Figures 2A and 2B). This finding stands in stark contrast to their failure to swarm or restrict fungal growth when in genetically homogeneous populations (Figures 2 and S2D–S2F). The restoration is significant, with the ability of the mixed population of knockout neutrophils to restrict fungal growth comparable to that of their wild-type counterparts (Figure 2C). Full restoration in swarming is also observed when cells from knockout mice were mixed 1:1 with their appropriate wild-type counterparts (Figures S2D–S2F). These results suggest that, when mixed, cells with defects at different steps along the LTB_4_ biosynthesis pathway can collaborate and compensate for their defects to restore their capacity to swarm and restrict fungal growth. We confirmed this finding using an enriched population of neutrophils (Figure S3), which matched those results obtained with bone marrow cells (Figure 2).

**Figure 3:**
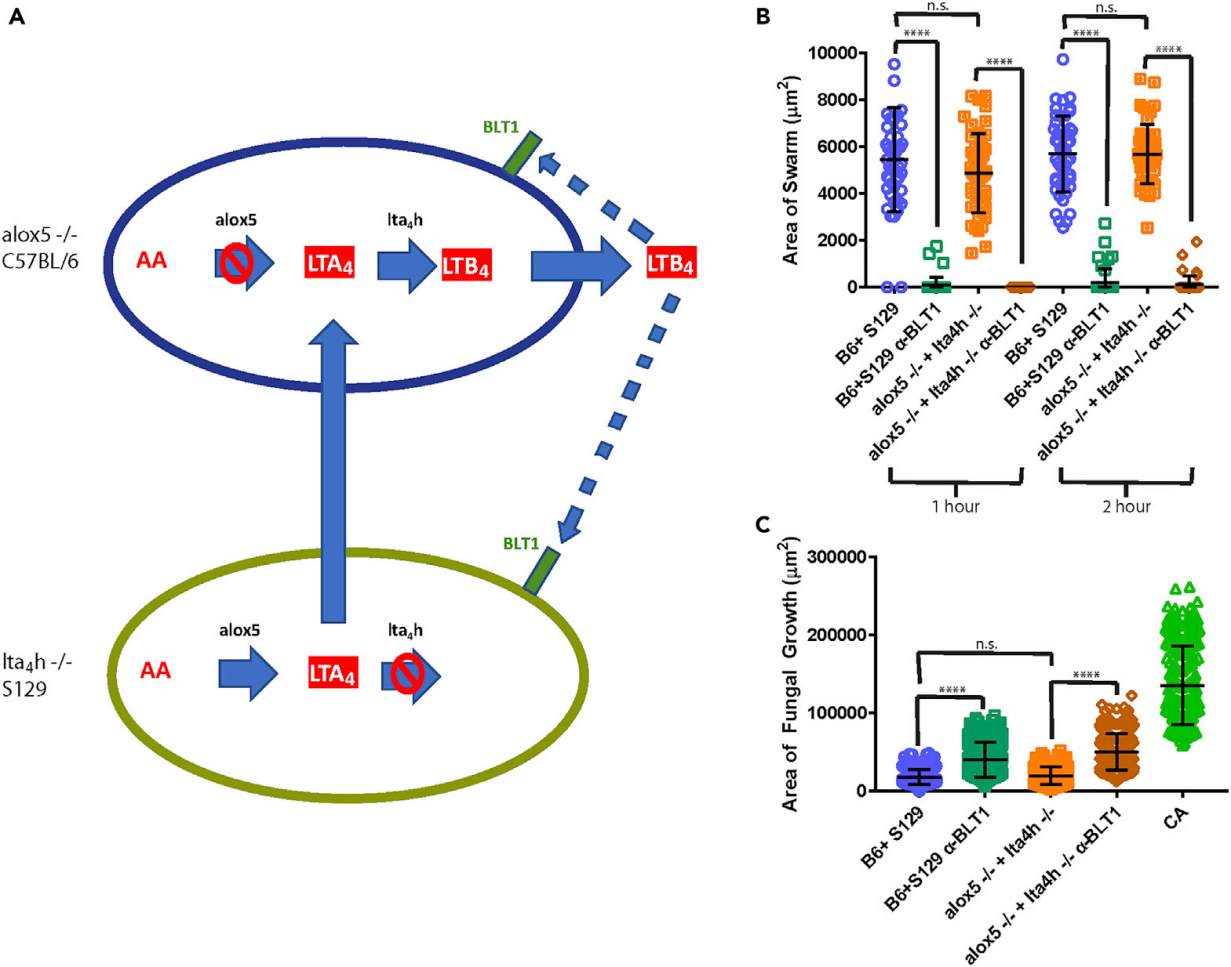
Transcellular synthesis is sufficient to restore swarming responses against *C. albicans* (A) A schematic diagram indicates the important steps in LTB_4_ synthesis, the block points for alox5−/− and lta_4_h−/− cell types, how they can collaborate by transcellular synthesis to create LTB_4_, and how LTB_4_ signals through BLT1 receptors present on both cell types to drive swarming. (B) Bone marrow cells of the indicated genotypes were pre-incubated with 10 μM of the BLT1 antagonist U-75302 or vehicle for 30 min and then added to the swarming assay. The area of the swarm was quantified at 1 and 2 h. N = 48 swarms across three independent experiments. (C) The area of fungal growth at the end of the assay (16 h) was also quantified. N≥286 swarms across three independent experiments. Mean and SD are shown. n.s. is non-significant and ∗∗∗∗p≤0.0001 by Kruskal-Wallis with Dunn’s post-test. See also Figure S2.

We hypothesized that the restoration of swarming in mixed knockout conditions might be due to transcellular biosynthesis of LTB_4_ (Figure 3A). According to this hypothesis, the *lta4h*^−/−^ neutrophils synthesize LTA_4_ and share this precursor with neighboring cells, of which the *alox5*^−/−^ could complete the synthesis and the release of the LTB_4_, which helps coordinate the activities of all mutant neutrophils possessing the BLT1 receptor. To directly test this hypothesis, we blocked LTB_4_ signaling using an antagonist of BLT1. In agreement with our previous results (Figure S2), disruption of the LTB_4_ signaling blocked swarming and reduced fungal restriction for the mixed wild-type cells (Figures 3B and 3C). Critically, blocking BLT1 signaling also disrupted swarming and fungal restriction for mixed knockout cells (Figures 3B and 3C). This result demonstrates that the restored ability of the mixed knockout cells to swarm is dependent on LTB_4_ signaling, suggesting that transcellular LTB4 biosynthesis is likely to occur when the knockout cells are mixed.

We measured LTB_4_ release during swarming by ELISA and found that the mixed combination of alox5^−/−^ and lta4h^−/−^ cells did release LTB_4_, consistent with an occurrence of transcellular synthesis (Figure S4A). The amount of LTB_4_ recovered from the mixed knockout population was less than that of LTB_4_ recovered from the mixed combination of their respective wild-type cells. However, the magnitude of the swarming responses and the ability to restrict fungal growth is effectively the same as wild-type levels (Figures 3B and 3C). These results suggest that the generation of LTB_4_ exclusively through transcellular biosynthesis of LTB_4_ is sufficient to drive robust swarming responses even though the amount of LTB_4_ generated appears to be less (Figures 2, 3 and S4A).

Paradoxically, a homogeneous population of *lta4h*^−/−^ cells appears to be producing low levels of LTB_4_ as measured by ELISA, despite lacking a critical biosynthetic enzyme for this process (Figure S4A). This observation was confirmed by ELISA with LTB_4_ assayed from supernatant following stimulation with calcium ionophore in the absence of *C. albicans*, suggesting that the presence of fungi and the possibility of a fungal source of LTA_4_H is not an explanation for the ELISA signal associated with *lta4h*^*−/−*^ neutrophils (Figure S4B). To better resolve whether this ELISA signal from *lta4h*−/− neutrophils is real, given its low level, the supernatant was also collected using an enriched population of neutrophils responding to live *C. albicans*, which once more confirmed the presence of this ELISA signal associated with *lta4h*^−/−^ neutrophils (Figure S4C). One potential explanation for why *lta4h*^−/−^ neutrophils generated a positive LTB_4_ ELISA signal is because, unlike alox5^−/−^ cells, *lta4h*^*−/−*^ cells remain capable of producing leukotriene A_4_ (LTA_4_). In the absence of LTA_4_H, LTA_4_ is rapidly converted non-enzymatically to inactive breakdown metabolites, including 6-trans-LTB_4_ (Haeggstrom, 2018). It is possible that LTA_4_ and breakdown metabolites are indistinguishable from LTB_4_ in this ELISA, and supernatant from *lta4h*^−/−^ neutrophils that produce LTA_4_ breakdown products yield a positive signal despite not containing any actual LTB_4_. In support of this interpretation, a bioactivity assay that takes advantage of the chemotactic potential of LTB_4_ (not shared by LTA_4_ breakdown metabolites) was developed and revealed that the supernatant from swarming chambers containing either *alox5*^−/−^ or *lta4h*^−/−^ neutrophils alone failed to elicit a response, consistent with a lack of true LTB_4_ production for *lta4h*^−/−^ neutrophils (Figure S4D). A significant increase in directed migration of neutrophils across a permeable Transwell was observed when supernatant was derived from a 1:1 mix of *alox5*^−/−^ and *lta4h*^−/−^, thereby revealing the presence of chemotactic bioactivity that was exclusively associated with the mixture of *alox5*^−/−^ and *lta4h*^−/−^ cells and this bioactivity likely represents LTB_4_ generated by transcellular means (Figure S4D).

**Figure 4:**
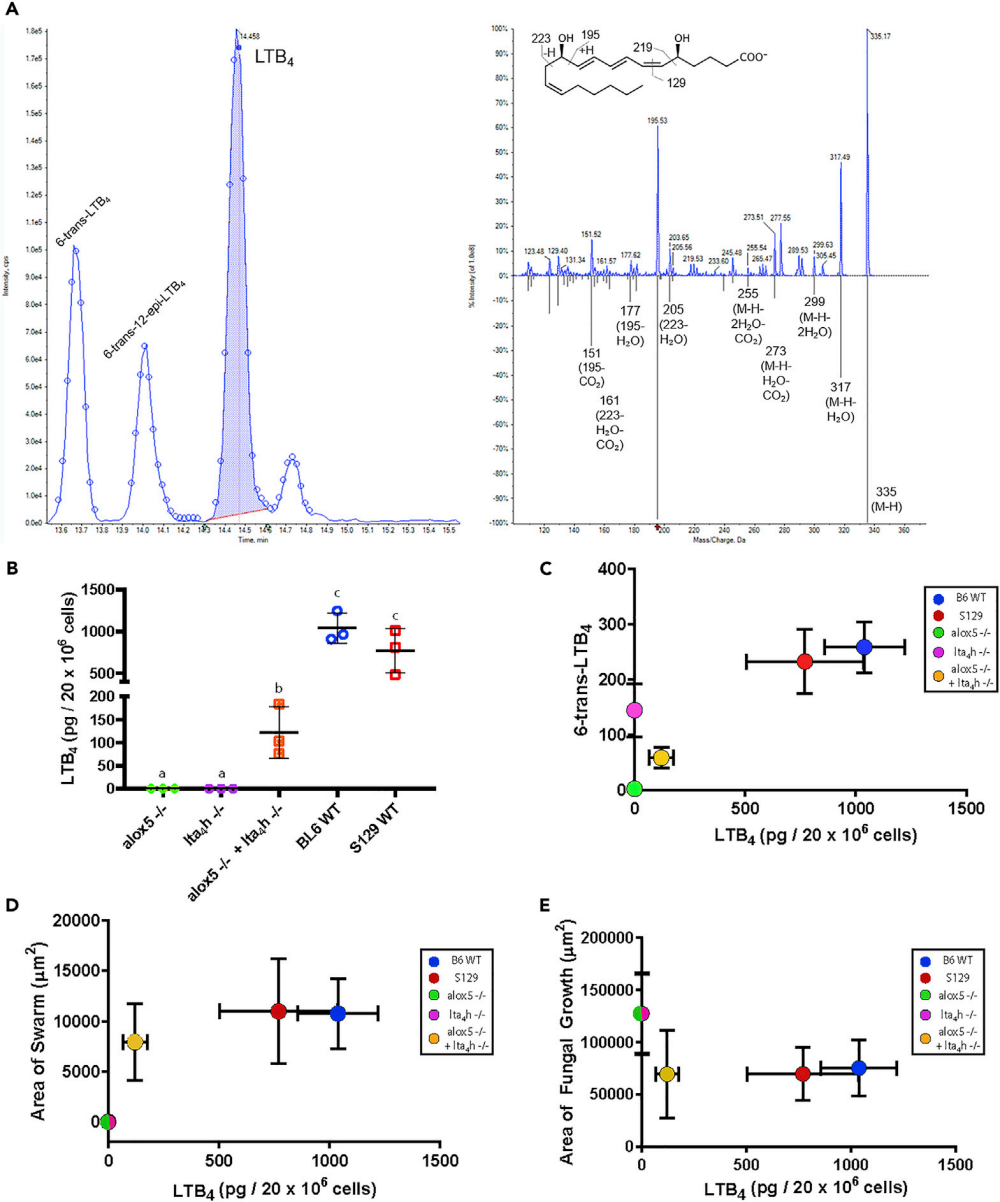
Heterogeneous mixtures of alox5^−/−^ and lta_4_h^−/−^ biosynthesize leukotriene B_4_ (A) LC-MS/MS targeted multiple reaction monitoring for *m/z* 335>195. The green arrows indicate the time interval of the quantitation (shaded). The selected data point denotes the time and intensity at which the spectrum, shown on the right, was recorded. *Right*, Enhanced product ion spectra of LTB_4_, top spectrum is from bone marrow cells, and the bottom spectrum is from the custom metabololipidomics library. The red arrow indicates Q3 (*m/z* 195). *Inset*, LTB_4_ structure with fragmentation. LTB_4_ fit from samples to LTB_4_ in custom metabololipidomics library (99.4%, see STAR Methods). (B) The amount of LTB_4_ detected in each condition was quantified. N= three independent experiments per group. (C–E) Dots represent the average of the indicated conditions, with error bars representing SD. (C) The amount of LTB_4_ is compared to the amount of 6-trans-LTB_4_ detected in each group. (D) The average area of a neutrophil swarm for each condition at 2 h was plotted against the amount of LTB_4_detected in the group. (E) The average area of fungal growth at 16 h for each condition was plotted against the amount of LTB_4_ detected in the group. Unshared letters represent significant differences between groups. p ≤ 0.05 by Student’s unpaired two-tailed t-test. See also Figures S3 and S4.

To further confirm that transcellular biosynthesis of LTB_4_ is occurring when mixing alox5^−/−^ and lta4h^−/−^ cells and to unambiguously clarify the nature of molecules detected by the ELISA, we conducted mass spectrometry on collected supernatants (Figure 4A and Table 1). Bone marrow cells derived from alox5^−/−^ mice failed to generate LTB_4_ and produced trace amounts of LTA_4_ non-enzymatic breakdown metabolites (Figures 4B, 4C and Table 1). Cells from lta4h^−/−^ mice also failed to generate LTB_4_ but did produce significant LTA_4_ and breakdown metabolites, 6-trans-LTB_4_, 6-trans-12-*epi*-LTB_4_, 5S,6S-diHETE, 5S,6R-diHETE as anticipated (Figures 4B, 4C and Table 1). The mixture of the *alox5*^−/−^ and *lta4h*^−/−^ cells resulted in the biosynthesis of LTB_4_ through transcellular processes as individual knockout neutrophils in isolation are incapable of generating LTB_4_ (Figures 4B, 4C and Table 1). In agreement with our ELISA results (Figure S4A), the amount of LTB_4_ produced in this condition of exclusively transcellular biosynthesis (*alox5*^−/−^ + *lta4h*^−/−^ neutrophils) is significantly lower than that produced during swarming by either wildtype cells (Figures 4B and 4C). Importantly, despite the difference in the magnitude of LTB_4_ biosynthesized, both swarming and fungal control are preserved and comparable between the mixed knockout and mixed wild-type counterpart conditions (Figures 3B, 3C, 4D and 4E).

**Table 1:** Quantification of lipid mediators released during swarming

	LTB_4_	6-trans-LTB_4_	6-trans-12-*epi*-LTB_4_	5S,6S-diHETE	5S,6R-diHETE
alox5^−/−^	0.0	2 ± 0.4	4 ± 0.6	0.0	0.0
lta_4_h^−/−^	0.0	144 ± 48	181 ± 62	50 ± 24	17 ± 20
alox5^−/−^ + lta_4_h^−/−^	121 ± 55	58 ± 19	41 ± 14	11 ± 4	7 ± 3
BL6 WT	1039 ± 181	258 ± 46	73 ± 3	27 ± 2	22 ± 1
S129 WT	770 ± 265	232 ± 58	81 ± 28	29 ± 9	19 ± 7
Media	0.0	0.0	0.0	0.0	0.0

Twenty million bone marrow cells from mice of the indicated genotypes were harvested and added to large swarming arrays of *C. albicans*. Supernatants and cells were harvested after 2 h and subjected to LC-MS/MS to examine lipid mediator biosynthesis. Concentrations represent picogram per 2 × 10^7^ cells and are the average ±SD of three independent experiments.

## Discussion

We measured mouse neutrophil swarming against *C. albicans* cluster targets and found that transcellular biosynthesis of LTB_4_ drives swarming responses that restrict the growth of fungi. Interfering with the LTB_4_ biosynthesis through deletion of key synthetic enzymes in *alox5*^−/−^ and *lta4h*^−/−^ mouse neutrophils and antagonizing LTB_4_ receptors disrupts swarming. Notably, the swarming of *alox5*^−/−^ mouse neutrophils cannot be restored by the addition of LTB_4_. These results reveal an essential role for the coordinated LTB_4_ release from neutrophils in accomplishing the swarming choreography. The dependence on coordinated LTB_4_ release distinguishes swarming from other 'traditional' neutrophil functions. For example, phagocytosis and ROS production are also altered when LTB_4_ biosynthesis is prevented in *alox5*^−/−^ mouse neutrophils, but, unlike swarming, phagocytosis and ROS production in these cells are restored by exposing the neutrophils to extrinsic LTB_4_, consistent with the previous reports (Miralda et al., 2017). Our study shows that swarming can only be restored when mixing *alox5*^−/−^ and *lta4h*^−/−^ neutrophils, where transcellular biosynthesis of LTB_4_ becomes possible. Furthermore, antagonizing LTB_4_ receptors disrupts swarming in these mixed cell experiments. These results highlight swarming as a unique and higher-order function of neutrophil coordination, which is more than simply the sum of activities manifested by individually functioning neutrophils.

Swarming, as an emergent neutrophil behavior, has been recently visualized in the context of mechanical (Alexander et al., 2020; Barros et al., 2021; Hopke et al., 2020; Knooihuizen et al., 2021; Yonker et al., 2021), thermal (Lammermann et al., 2013), or infected wounds (Chtanova et al., 2008) in mice and zebrafish. For human neutrophils, *ex vivo* testing revealed disrupted swarming in patient populations at risk for fungal infections, e.g., transplant recipients, cirrhosis, trauma, chronic granulomatous disease, cystic fibrosis, etc. (Barros et al., 2021; Hopke et al., 2020; Knooihuizen et al., 2021; Yonker et al., 2021). Furthermore, in a single patient case study, we found that restoring neutrophil swarming correlated with reduced numbers of infections experienced by that patient (Alexander et al., 2020). In parallel efforts, lipid mediators are increasingly understood to be consequential in various pathological processes, and targeting biosynthesis may have therapeutic benefits in these circumstances (Haeggstrom, 2018). The range of conditions that could be corrected by the manipulation of lipid mediator levels spans from common infections (Jordan and Werz, 2021) to complex conditions like Alzheimer’s disease (Emre et al., 2022).

Our understanding of transcellular biosynthesis of lipid mediators in homogeneous cell populations benefits from earlier studies in heterogeneous mixtures of neutrophils with other cell types (Fabre et al., 2002). Transcellular biosynthesis helps coordinate the activity of immune and non-immune cells sharing the same space, e.g., neutrophils, lymphocytes, platelets, and endothelial cells (Claesson and Haeggstrom, 1988; Fiore and Serhan, 1990; Marcus et al., 1982; Odlander et al., 1988; Serhan et al., 1984a, 1984b, 2020). Transcellular biosynthesis is facilitated by the proximity of two distinct cell types that individually lack but collectively express all necessary enzymes to synthesize a particular mediator (Corey et al., 1980). One of the eicosanoid intermediates that is most shared among immune and non-immune cells is LTA_4_, produced and released in large amounts by neutrophils (Afonso et al., 2012; Fiore and Serhan, 1989). When LTA_4_ is taken up by endothelial cells, keratinocytes, erythrocytes, or alveolar macrophages, which express LTA_4_ hydrolase, these cells can biosynthesize LTB_4_ (Dieterle et al., 2020). An indication that transcellular biosynthesis is likely to be quite common is the observation that close to half of the LTA_4_ produced by neutrophils is released extracellularly rather than converted to LTB_4_ (Claesson and Haeggstrom, 1988; Fiore and Serhan, 1990; Marcus et al., 1982; Odlander et al., 1988; Serhan et al., 1984a, 1984b, 2020).

Our study raises several important questions that will be addressed in future studies. It is not fully understood how transcellular biosynthesis intermediates are transported between neutrophils. For transcellular biosynthesis of LTB_4_ to occur, LTA_4_ must be passed from *lta4h*^−/−^ neutrophils, which have functional 5-LOX, to the *alox5*^−/−^ neutrophils, which have functional LTA_4_ hydrolase. By collaborating in this fashion, the *alox5*^−/−^ and *lta4h*^−/−^ cells can produce functional LTB_4_, which can then be released to drive the recruitment and swarming of both cell types. LTA_4_ has a short half-life (Fiore and Serhan, 1989; Haeggstrom, 2018; Stsiapanava et al., 2017) and is likely hydrolyzed immediately after release (Fiore and Serhan, 1989). Transportation modes that increase the biological half-life of LTA_4_ should be considered. For example, associations of LTA_4_ to lipid membranes (Fiore and Serhan, 1989) and to chaperone molecules, like albumin (Fitzpatrick et al., 1982), have been proposed to protect LTA_4_ in the extracellular space between various cell pairs. A transport mediated by exosomes has also been suggested for shuttling LTB_4_ from neutrophils to other neutrophils (Dieterle et al., 2020) and may also be applicable to LTA_4_. This mechanism may also be consistent with the relay model of neutrophil signaling during swarming (Dieterle et al., 2020).

### Limitations of the study

Much of this work was conducted with bone marrow cells that feature 40% or less mature neutrophils within the total cell population. It is, therefore, possible that other cells within the bone marrow may influence the swarming observed herein. Our observations were confirmed using enriched neutrophil populations (65–75% mature neutrophils) from bone marrow. Nevertheless, further work is needed to exclude potential influences of the non-neutrophil cellular component within the bone marrow.

The relevance of our findings in mice to human neutrophils remains to be examined. Human neutrophils display multiple levels of redundancy in swarming, with additional factors besides LTB_4_, like IL-8 and complement factors, partially compensating for the loss of LTB_4_. Unlike human neutrophils, LTB_4_ appears to be the only driving factor of neutrophil swarming in mice (Hopke et al., 2020; Kienle et al., 2021; Lammermann et al., 2013; Reategui et al., 2017). Our study demonstrates that transcellular LTB_4_ biosynthesis is necessary and sufficient to orchestrate swarming and restriction of fungal growth by a mixture of genetically deficient mouse neutrophils that are individually incapable of completing LTB_4_ synthesis. We suggest that transcellular LTB_4_ biosynthesis is likely to be important in orchestrating wild-type mouse neutrophil swarming as well. Transcellular LTB_4_ biosynthesis is facilitated by the large proportion of LTA_4_ released from wild-type neutrophils as revealed by detection of LTA_4_ non-enzymatic breakdown metabolites (Afonso et al., 2012; Fiore and Serhan, 1989). Future investigations are necessary to characterize the role of LTB_4_ transcellular biosynthesis in human and mouse swarming and its contribution to neutrophil-mediated host defense following sterile injury and infection.

## STAR★Methods

### Key resources table

**Table:** 

REAGENT or RESOURCE	SOURCE	IDENTIFIER
**Antibodies**		

Mouse monoclonal PE anti-human CD45	Biolegend	Cat#304058; RRID: AB_2564156
Mouse monoclonal PerCP-Cy5.5 anti-human CD16	Biolegend	Cat#302028; RRID: AB_893263
Mouse monoclonal APC anti-human CD66b	Invitrogen	Cat#17-0666-42; RRID: AB_2573152
Rabbit polyclonal Alexa fluor 488 anti-human BLT1	Bioss	Cat# bs-2654R-A488; RRID: AB_2924305
Mouse monoclonal anti-human CD16/32	Invitrogen	Cat#14-0161-82; RRID: AB_467133
PE anti-mouse Ly-6G Antibody, clone 1A8	BioLegend	Cat#127608; RRID: AB_1186099

**Chemicals, peptides, and recombinant proteins**		

U-75302	Cayman Chemicals	70705; CAS 119477-85-9
SYTOX Green	ThermoFisher Scientific	S7020
Leukotriene B4	Cayman Chemicals	20110;CAS 71160-24-2
Dihydrorhodamine 123	ThermoFisher Scientific	Cat#D23806
Cytochalasin D – from *Zygosporium mansonii*	Sigma-Aldrich	Cat#C8273
Poly-L-Lysine solution- 0.1% in H2O	Sigma-Aldrich	P8920-100ML
d_4_-Leukotriene B_4_	Cayman Chemicals	Cat#320110
Methanol, Optima LC/MS Grade	Thermo Fisher Scientific	Cat#A456-4
OmniSolv Hexanes 64% n-hexane For HPLC, Spectrophotometry and Gas Chromatography	VWR	Cat#HX0296P-1
Methyl Formate	Sigma-Aldrich	Cat#259705-2L
Water, Optima LC/MS Grade	Thermo Fisher Scientific	Cat#W6-4
Formic Acid (ACS Reagent, ≥96%)	Sigma-Aldrich	Cat#695076-500ML
6-trans-Leukotriene B_4_	Cayman Chemicals	Cat#35250
6-trans-12-epi-Leukotriene B_4_	Cayman Chemicals	Cat#35265
5*S*,6*R*-diHETE	Cayman Chemicals	Cat#35200
5*S*,6*S*-diHETE	Cayman Chemicals	Cat#35210

**Critical commercial assays**		

Leukotriene B4 ELISA kit	Cayman Chemicals	520111
Mouse Myeloperoxidase DuoSet ELISA	R&D systems	Cat#DY3667
EasySep Mouse neutrophil enrichment kit	Stemcell	Cat#19762

**Experimental models: Organisms/strains**		

*Candida albicans* SC5314 iRFP	Robert Wheeler	Hopke et al. 2016
*Mus musculus* C57BL/6J	The Jackson Laboratory	Strain #: 000664
*Mus musculus* 129S1/SvImJ	The Jackson Laboratory	Strain #:002448
*Mus musculus* B6.129S2-Alox5^tm1Fun^/J	The Jackson Laboratory	Strain #:004155
*Mus musculus* 129-Lta_4_h^tm1Bhk^/J	The Jackson Laboratory	Strain #:004446

**Software and algorithms**		

FlowJo	NA	https://www.flowjo.com/
ImageJ	NA	https://imagej.nih.gov/ij/
Analyst version 1.7.1	Sciex	https://sciex.com/support/software-support/software-downloads
LibraryView version 1.4	Sciex	https://sciex.com/support/software-support/software-downloads
Sciex OS-Q version 1.7.0.36606	Sciex	https://sciex.com/support/software-support/software-downloads

**Other**		

PolyPico PicoSpotter	NA	https://www.polypico.com/products/
Isolute C18 (100mg/3mL) columns	Biotage	Cat#220-0010-B
Kinetex C18 column (100 mm × 3.0 mm x 2.6 μm, 100Å)	Phenomenex	Cat#00D-4759-Y0
6500+ Triple Quadrupole QTRAP mass spectrometer	Sciex	Cat#5062192C, https://sciex.com/products/mass-spectrometers/qtrap-systems/qtrap-6500plus-system
ExionLC AC System	Shimadzu/Sciex	Cat#5036665, https://www.sciex.com/cr/products/hplc-products/exionlc
Extrahera automated extractor	Biotage	Cat#414001, https://www.biotage.com/automated-solid-phase-extraction

### Resource availability

#### Lead contact

Further information and requests for resources and reagents should be directed to and will be fulfilled by the lead contact, Bryan Hurley (bphurley@mgh.harvard.edu).

#### Materials availability

This study did not generate any new unique reagents. Slides and plates for the swarming assays are available through the BioMEMS Core at the Massachusetts General Hospital https://researchcores.partners.org/biomem/about.

### Experimental model and subject details

#### Animals

The following strains of mice were obtained from Jackson Laboratories: wild-type C57BL/6J and S129 (129S1/SvImJ), knockout mice alox5^−/−^ (B6.129S2-Alox5^tm1Fun^/J)(Chen et al., 1994) and lta_4_h^−/−^(129-Lta_4_h^tm1Bhk^/J) (Byrum et al., 1999). Eight to twenty weeks old male and female mice of different genotypes were used to isolate bone marrow cells. The Institutional Animal Care and Use Committee at Massachusetts General Hospital (MGH) approved the animal protocols used in this study. The mice were housed and bred in the the animal facility of MGH. The laboratory animal care and use program at MGH is accredited by AAALAC International, has an assurance with the Office of Laboratory Animal Welfare (OLAW) and is registered with the United States Department of Agriculture (USDA).

#### Microbial strains

*Candida albicans* SC5314 far-red fluorescence expressing strain (SC5314 iRFP) was a kind gift of Robert Wheeler at the University of Maine. (Hopke et al., 2016) *C. albicans* was inoculated to fresh liquid YPD and grown overnight with shaking at 30°C.

### Method details

#### Isolation of bone marrow cells and purification of bone marrow neutrophils

Bone marrow cells were isolated from C57BL/6, 129S1, alox5^−/−,^ and lta_4_h^−/−^ mice (Jackson Laboratories) as described previously with mild modifications (Boxio et al., 2004). Briefly, mice were euthanized with CO_2,_ and femurs and tibia were flushed with HBSS without calcium and magnesium (Thermo Fisher Scientific). Spicules or bone matrix were removed by 40 μm cell strainer (Fisher). Red blood cells were lysed in cold NH_4_Cl lysis buffer as described previously. (Hurley et al., 2004) About 1.5 – 2.5 × 10^7^ bone marrow cells were isolated per mouse and 26–40% of the bone marrow cells were CD11b+Ly6G+ neutrophils as confirmed by flow cytometry by using fluorescently-labeled antibodies for CD45, CD11b and Ly6G (Thermo Fisher Scientific). The majority of neutrophils (94%) were morphologically mature and functionally competent, as reported previously. (Boxio et al., 2004) This technique allows for rapid isolation of 17-fold more neutrophils than those isolated from peripheral blood per mouse. Additional neutrophil purification was performed using the EasySep mouse neutrophil enrichment kit (STEMCELL), following the manufacturer’s recommended protocol. The purity of CD11b+Ly6G+ neutrophils was 65-75% as evaluated by flow cytometry analysis.

#### Phagocytosis and ROS production during *C. albicans* challenge

Murine neutrophils were harvested from the tibias and femurs of B6 and alox5^−/−^ mice as previously described (Wang et al., 2006). Briefly, bones were crushed in FACs buffer (2% heat-inactivated fetal bovine serum in PBS), strained through a 40 μM filter, and red blood cells were lysed using 0.2% and 1.6% NaCl solutions. Neutrophils were then harvested by a Ficoll gradient (Histopaque, Sigma Aldrich). To assess phagocytosis and ROS production, neutrophils were co-incubated with a far-red fluorescent protein-expressing *C. albicans* strain (Hopke et al., 2016) at a ratio of 5 yeast cells per neutrophil for 1 h at 37°C in a 1.5-mL tube. Samples were incubated with dihydrorhodamine-1,2,3 (DHR-123 at 1 μM, Life Technologies, Eugene, OR) to assess ROS production for each condition. Where appropriate, neutrophils were treated with 30 μM of cytochalasin-D (Sigma) to inhibit phagocytosis or with LTB_4_ (0.6 nM) for phenotype rescue. Following co-incubation, samples were placed on ice and labeled with Ly6G-PE (BioLegend) for 15 min, washed in FACs buffer, and plated in a 96-well U-bottom plate. A BD FACSCeleta Cell Analyzer (BD Biosciences) with a high-throughput plate adaptor running BD FACSDiva Software (v9.0). Percent ROS was measured by selecting doubly positive Ly6G-PE neutrophils and DHR-123 fluorescent cells, whereas percent phagocytosis was measured by fluorescent *C. albicans* in neutrophils. Flow data were analyzed using FlowJo 10 software (FlowJo, Ashland, OR).

#### Swarming array printing

Utilizing a microarray printing platform (Picospotter PolyPico, Galway, Ireland), we printed a solution of 0.1% poly-l-lysine (Sigma-Aldrich) and ZETAG targets with 100 μm diameter. For microscopy and ELISA experiments, we printed eight by eight arrays in a sixteen-well format on ultra-clean glass slides (Fisher Scientific). For LC-MS/MS experiments, we printed over 4500 targets covering the glass slide. Slides were screened for accuracy and then dried at 40°C for 2 h on a heated block. After 2 h, slides were removed from the heat block and left at room temperature until required.

#### Patterning of *Candida albicans* cluster targets

Swarming arrays were created as described (Hopke et al., 2020). Briefly, 16-well ProPlate wells (Grace Bio-labs) or single-well ProPlate wells were attached to glass slides with printed arrays of poly-l-lysine/ZETAG. A suspension of the desired target, in this case, live *C. albicans*(SC5314 iRFP) yeast in water, was added to each well (50 uL per well for the 16-well format, 1.5 mL for the single well) and incubated with rocking for 5 min. Following incubation, wells were thoroughly washed out with PBS to remove unbound targets from the glass surface. Wells were screened to ensure appropriate patterning of targets onto the spots with minimal non-specific binding before use.

#### Swarming experiments

All imaging experiments were conducted using a fully automated Nikon TiE microscope. Time-lapse imaging was conducted using a 10x Plan Fluor Ph1 DLL (NA = 0.3) lens, and endpoint images were taken with a 2x Plan Apo (NA = 0.10) lens. Swarming targets (*C. albicans* clusters) to be observed during time-lapse were selected and saved using the multipoint function in NIS elements prior to loading of cells. Bone marrow cells or enriched bone marrow neutrophils were stained with Hoechst (Thermo Fisher Scientific) and resuspended in IMDM with 20% FBS (Thermo Fisher Scientific). 500,000 cells were added to each well for individual genotype conditions. 250,000 cells each were added in mixed genotype conditions. All selected points were optimized using the Nikon Perfect Focus System before launching the experiment. In experiments using chemical inhibitors, neutrophils were pre-incubated with the chemical or appropriately matched vehicle control for 30 min before use. The supernatants were collected 2 h after the cells were added and saved at −80°C after removing the cells by centrifugation.

#### Image analysis

Area analysis was performed manually by outlining the swarms or areas of fungal growth in the NIS-elements (v4.00.12; Nikon Inc) or FIJI (FIJI is just ImageJ v2.0.0-rc-59/1.52p, NIH) software. For the area of the swarm, only the swarm itself (just the immune cells) was measured. This was done using the DAPI fluorescent channel image, using Hoechst staining to identify neutrophils. For areas of fungal growth, a combination of brightfield and fluorescent channels was used. Fungi used in experiments were always far-red fluorescent (Hopke et al., 2016). We combined the appropriate fluorescent channel with the brightfield image to be sure we included any escaped fungal elements, like lone hyphae, that may not show up well in the fluorescent channel.

#### Bone marrow cell culture

Bone marrow cells from single or mixed cell types were seeded in 96-well round-bottom tissue culture plates at 200 μL/well with 5×10^6^ cells/mL. Cells were incubated with calcium ionophore A23187 (Sigma-Aldrich) at 20 μg/mL at 37°C with 5% CO_2_ for 1 h. Cells were removed by centrifugation at 500 ×g for 5 min. The supernatants were saved at −80°C for LTB_4_ ELISA assays.

#### LTB_4_ quantification by ELISA

Supernatants from the swarming assay for each condition were collected at the indicated time points and subjected to a competitive LTB_4_ ELISA (Cayman chemical) according to the manufacturer’s protocol. Briefly, 50 μL LTB_4_ standards diluted in 1:2 series and supernatants from the swarming assay were added to the 96-well plate precoated with mouse anti-rabbit IgG and incubated with LTB_4_ antiserum and AChE linked to LTB_4_ (tracer) at 4°C overnight. The plate was then washed five times with wash buffer, followed by incubation with Ellman’s reagent for 90–120 min. The absorbance at 405 nm was measured by SpectraMax iD5 microplate reader (Molecular Devices). The readings of diluted standards were plotted as logit B/B_0_ versus log LTB_4_ concentration using a linear fit and were used to determine sample LTB_4_ concentrations according to the manufacturer’s instructions.

#### BLT1 receptor quantification on mouse neutrophils

Bone marrow cells isolated from C57BL/6, alox5^−/−^, S129, and lta_4_h^−/−^ mice were applied to LIVE/DEAD fixable Dead Cell staining by incubating with Near-IR fluorescent reactive dye (Thermo Fisher Scientific) in HBSS at room temperature for 15min in the dark, followed by two washes with HBSS. The cells were then resuspended in eBioscience^TM^ Flow Cytometry staining buffer (Thermo Fisher Scientific) and incubated with rat anti-mouse CD16/CD32 monoclonal antibody on ice for 10 min to block the Fc receptor. The following antibodies were then added to stain the cells on ice for 15 to 30 min in the dark: PE-conjugated rat anti-mouse CD45 (BioLegend), PerCP-Cyanine5.5-conjugated rat anti-mouse CD11b (Thermo Fisher Scientific), APC-conjugated rat anti-mouse Ly-6G (Thermo Fisher Scientific), Alexa Fluor 488-conjugated rabbit anti-mouse BLT1 (Bioss Antibodies). The cells were washed and applied to flow cytometry analysis using Attune NxT Flow Cytometer (Thermo Fisher Scientific). The data were analyzed by FlowJo. Neutrophils were gated as CD45^+^CD11b+Ly-6G+ live cells and the mean fluorescence intensity (MFI) of BLT1 on neutrophils was compared between C57BL/6, alox5^−/−^, S129, and lta_4_h^−/−^ genotypes.

#### Chemotaxis assay

The chemoattractive activity of the supernatants obtained from swarming experiments was measured by performing a bone marrow cell transmigration assay using 96-well Transwell with a pore size of 3μm (Corning). Bone marrow cells were isolated from the femoral and tibial bones of C57BL/6J alox5^−/−^ or lta_4_h^−/^ mice, as described above. One hundred microliters of supernatant were added to the bottom well, and 10^6^ bone marrow cells in 75 μL HBSS were added to the inside of the Transwell insert. After incubation at 37°C for 2 h, the inserts were removed. Bioactivity was determined by the number of neutrophils that migrate through the Transwell towards the conditioned swarming supernatant or LTB_4_ (0.2 ng/mL). Moreover, MPO assay was performed with the cells migrated to the bottom wells as described previously. (McCormick et al., 1995)

#### Targeted liquid chromatography-tandem mass spectrometry metabololipidomics

Bone marrow cells (20 × 10^6^ cells/group) from alox5^−/−^, lta_4_h^−/−^, alox5^−/−^ + lta_4_h^−/−^, C57BL/6J wild-type, or 129S1 wild-type suspended in 2 mL IMDM (phenol red free) containing 0.1% human serum albumin were incubated for 2 h at 37°C in glass slides/wells covered in large arrays of *C. albicans* clusters (100 μm in diameter). The incubations were quenched with 2 mL 100% ice-cold LC-MS grade methanol (Thermo Fisher Scientific, Waltham, MA, USA) containing 500 pg d_4_-LTB_4_ (Cayman Chemicals, Ann Arbor, MI, USA) for calculating recovery and quantity of endogenous materials. Protein was precipitated by storage at −80°C for a minimum of 30 min, followed by centrifugation at 1000 ×*g* for 10 min at 4°C. Supernatants were extracted using an automated extractor (Extrahera, Biotage, Charlotte, NC, USA) by solid phase extraction on 100 mg C18 columns (Biotage) as described in (Shay et al., 2021). LTB_4_ and its isomers (6-trans-LTB_4_, 6-trans-12-epi-LTB_4_, 5*S*,6*S*-diHETE, and 5*S*,6*R*-diHETE) were eluted with spectrophotometric grade methyl formate (Sigma-Aldrich). Samples were evaporated under a gentle stream of nitrogen gas and immediately resuspended in a LC-MS grade methanol-water mixture (50:50, *v/v*) for analysis by a 6500^+^ Triple Quadrupole QTRAP mass spectrometer in low mass and negative polarity mode (Sciex, Framingham, MA, USA) equipped with an ExionLC (Shimadzu, Kyoto, Japan). A Kinetex C18 column (100 mm × 3.0 mm × 2.6 μm, 100 Å, Phenomenex) was maintained at 50°C in a column temperature-controlled oven.

LTB_4_ and its isomers (6-trans-LTB_4_, 6-trans-12-epi-LTB_4_, 5*S*,6*S*-diHETE, and 5*S*,6*R*-diHETE) were quantified using targeted multiple reaction monitoring (MRM) with the following settings: Q1 (*m/z*) = 335.2, Q3 (*m/z*) = 195.1 or 115.1 (diHETEs), declustering potential = −40 V, entrance potential = −10 V, collision energy = −22 V, and collision cell exit potential = −12 V. The data were acquired with Analyst version 1.7.1 (Sciex) and analyzed with, and screen captured from Sciex OS-Q version 1.7.0.36606 (Sciex). The LTB_4_ calibration curve was used for quantification and had a correlation coefficient (r^2^) of 0.99742. The solvents, gradient, MRM, and enhanced product ion (EPI) mode settings are detailed in (Shay et al., 2021). Each mediator was identified by a matching retention time, and an unbiased MS/MS library fit (≥70%) to synthetic materials, as well as the presence of key structural fragments. The synthetic materials in the custom metabololipidomics library were validated against authentic materials and was created using LibraryView version 1.4 (Sciex). The following library smart confirmation search parameters were used for the identification of LTB_4_ and its isomers: precursor mass tolerance ±0.8 Da, fragment mass tolerance ±0.8 Da, collision energy ±5 eV, use polarity, an intensity threshold of 0.02, a minimum purity of 5.0%, and an intensity factor of 100.

### Quantification and statistical analysis

All statistics were conducted using GraphPad Prism 7.03 software. Data were tested for normality using a D’Agostino-Pearson omnibus normality test. Normally distributed data were analyzed with Student’s T-test or One Way ANOVA with Tukey’s post-test. Non-normally distributed data were analyzed with a Mann-Whitney or Kruskal-Wallis with Dunn’s post-test where appropriate. Statistical significance was considered for p < 0.05 and is provided in the relevant figure legends. Error bars represent standard deviation unless otherwise indicated.

## Data Availability

• All data reported in this article will be shared by the lead contact on request. • This article does not report any original code. • Any additional information required to reanalyze the data reported in this article is available from the lead contact on request.
