# Supplementary material for: Transcellular biosynthesis of leukotriene B4 orchestrates neutrophil swarming to fungi
Source: iScience. 2022 Sep 28;25(10):105226. doi: 10.1016/j.isci.2022.105226 (PMC9576560; doi:10.1016/j.isci.2022.105226)
Supplement: Document S1. Figures S1–S4 [file mmc1.pdf]

iScience, Volume *25*

Supplemental information

Transcellular biosynthesis of leukotriene B_4 orchestrates neutrophil swarming to fungi

Alex Hopke, Tian Lin, Allison K. Scherer, Ashley E. Shay, Kyle D. Timmer, Brittany Wilson-Mifsud, Michael K. Mansour, Charles N. Serhan, Daniel Irimia, and Bryan P. Hurley

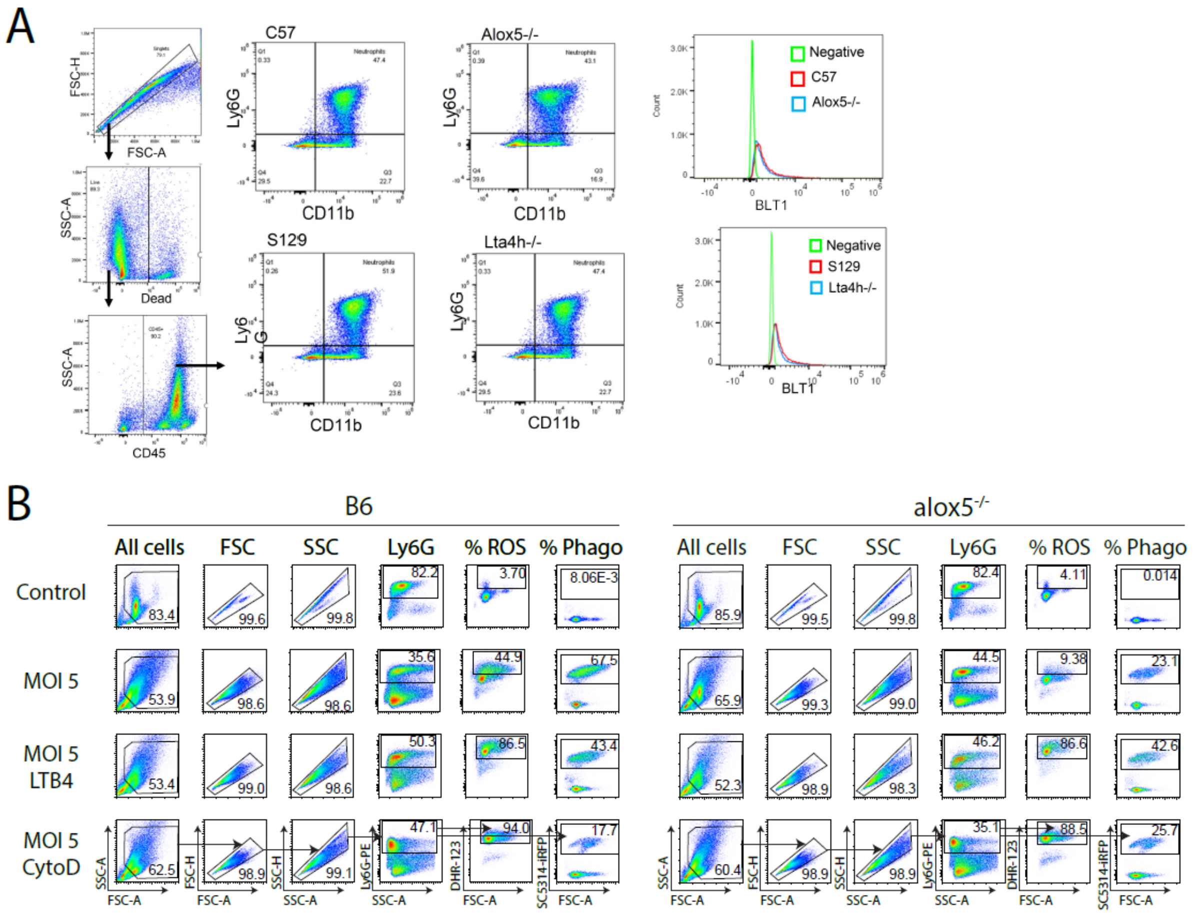

Figure S1: Flow cytometry analysis of BLT1 expression, phagocytosis and ROS production in knockout and the background wild-type bone marrow neutrophils (C57 and alox5-/- , S129 and lta_4h-/-), Related to Figure 1. (A) Isolated bone marrow cells were stained with fluorophore-conjugated CD45, CD11b, Ly6G and BLT1 antibodies and applied to flow cytometry analysis. Live CD45+ leukocytes were gated to analyze BLT1 expression on CD11b+Ly6G+ neutrophils. BLT1 expression was compared on neutrophils from C57 and alox5-/- or S129 and lta_4h-/. **(B)** Mouse neutrophils were harvested from the bone marrow of B6 and alox5$^{-/-}$ mice and challenged with live *C. albicans* expressing a far-red fluorescent protein. Representative flow plots of the gating scheme used to measure the percentage of neutrophils producing ROS, by dihydrorhodamine-1,2,3 (DHR-123) fluorescence, and the percentage of neutrophils phagocytosing *C. albicans* (by far red fluorescence) are shown. Neutrophils were incubated with *C. albicans* at a multiplicity of infection (MOI) of 5 with vehicle, LTB_4 (0.6 nM) or CytoD (30 µM). Cells were then stained, and Ly6G-PE positive neutrophils were selected from FSC and SSC gating. Subsequently, neutrophils were quantified for DHR-123 fluorescence and for fluorescent *C. albicans*.

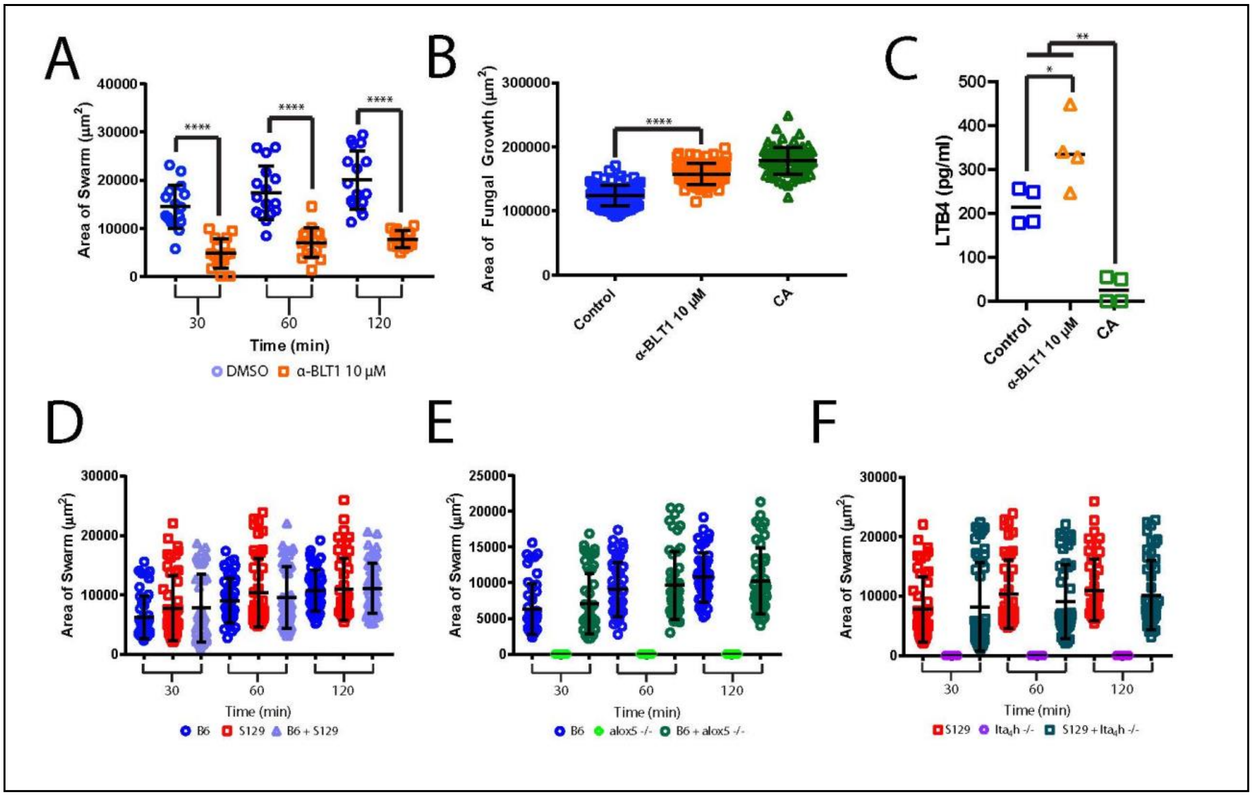

Figure S2: Reduced swarming against live *C. albicans* clusters by wild type mouse bone marrow cells after incubation with the BLT1 blocker U75302, Related to Figure 2 and Figure 3. (**A**)The dynamics of swarming over time was quantified. N= 16 swarms from one experiment. (**B**)The amount of fungal growth present at 16 hours was then quantified to examine how effectively the swarms restrict fungi. N≥ 79 swarms from one experiment. (**C**) Supernatants from the swarms were collected at 1 hour and subjected to ELISA to examine LTB_4 production. N= 4 replicate wells from two experiments. (**D-F**) Bone marrow cells were harvested from mice of the indicated genotypes and added to the swarming arrays either individually or as part of the indicated mixture, either wild types (**D**), the alox5$^{-/-}$ and its respective wild type (**E**) or lta$_4$h$^{-/-}$ and its respective wild type (**F**), at a ratio of 1:1. N=48 swarms across three independent experiments.

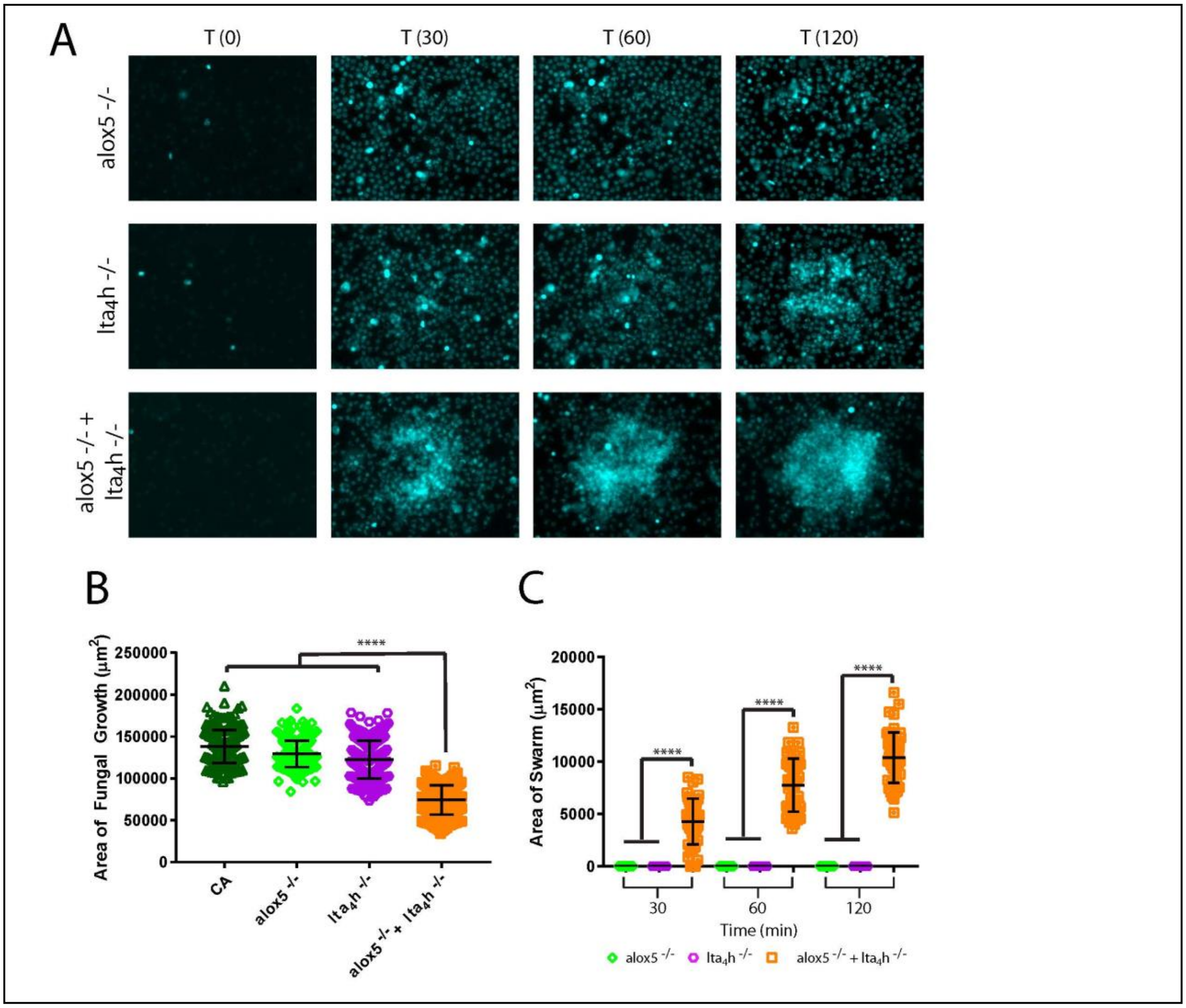

Figure S3: Mouse neutrophil swarming against live *C. albicans* clusters, Related to Figure 2. Cells from the bone marrow of mice from the indicated genotypes were further purified by magnetic separation to create an enriched PMN population prior to addition to swarming arrays. Fluorescent timelapse microscopy was used to visualize (**A**) Hoechst-stained neutrophils and follow the dynamics of swarming, with representative images shown. Fungal growth was quantified 16 hours after the start of the assay. (**B**) The area of fungal growth in the presence of homogenous alox5$^{-/-}$ and lta$_4$h$^{-/-}$ neutrophils was comparable to that in no-neutrophil control (C*andida* alone, CA). The area of fungal growth was significantly reduced in the presence of a heterogenous mixture of alox5$^{-/-}$ and lta$_4$h$^{-/-}$. N= 240 swarms across three independent experiments. (**C**) The area of the swarm at *C. albicans* targets was quantified at the indicated times. N= 48 swarms across three independent experiments. ****p≤0.0001 by Kruskal-Wallis.

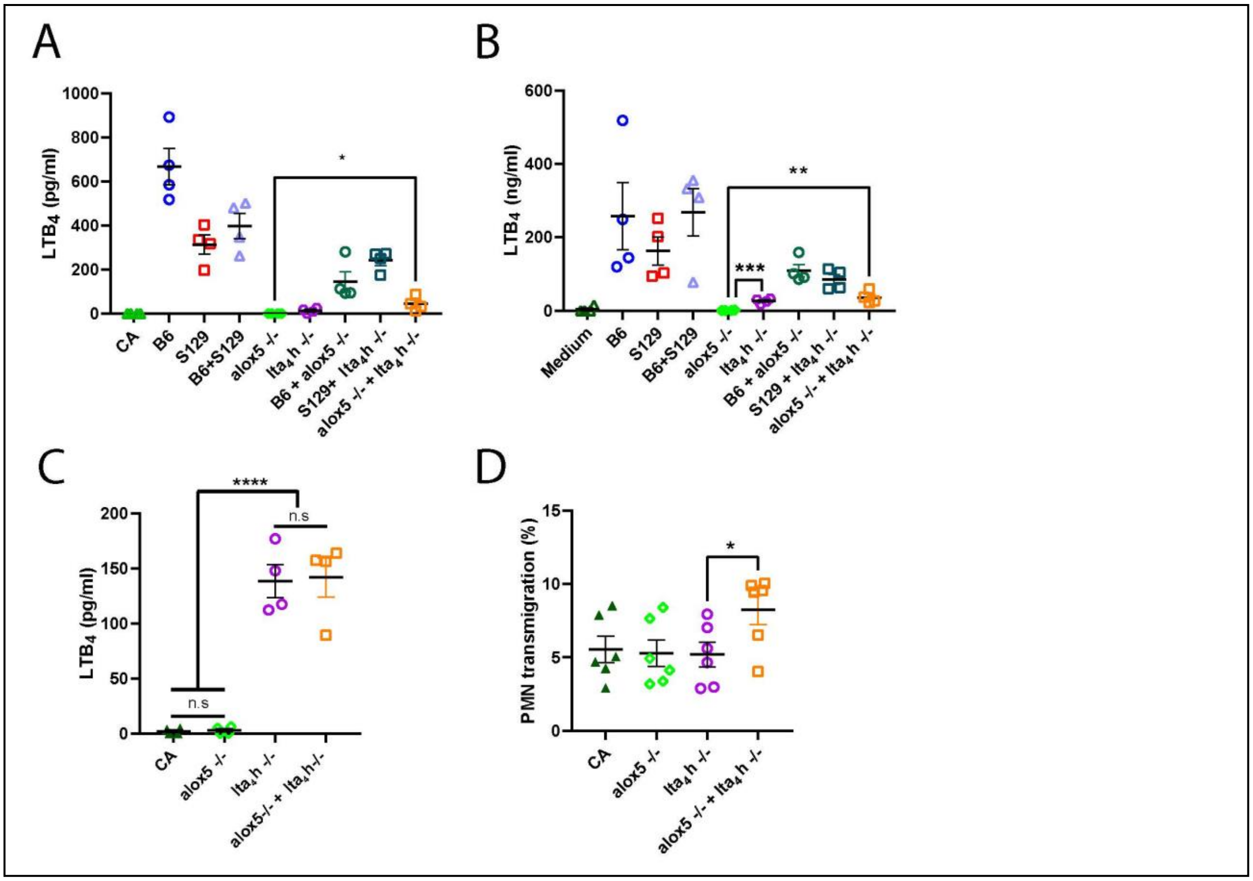

Figure S4: LTB_4 release by mouse neutrophils, Related to Figure 3 and Figure 4 (**A**) Supernatants from swarming experiments conducted with bone marrow cells were harvested at 2 hours and subjected to ELISA to measure LTB_4 levels. N= 4 in representative experiment out of 3 independent experiments yielding similar results. (**B**) Bone marrow cells of mice from the indicated genotypes were incubated alone or mixed at a ratio of 1:1 with calcium ionophore and supernatants were subjected to ELISA to quantify LTB_4. N= 2 across two independent experiments. (**C**) Supernatants from swarms conducted with enriched neutrophils were harvested at 2 hours and subjected to ELISA to examine LTB_4 levels. N= 4 in representative experiment out of two independent experiments yielding similar results. (**D**) Supernatants from the swarms of enriched neutrophils were also used in a transwell chemotaxis assay. The number of transmigrating neutrophils was quantified and presented as a percentage of the total. N= 6 supernatants run per condition, collected across three independent experiments. * p≤0.05, ** p≤0.01, *** p≤0.001, ****p≤0.0001 by Students unpaired two-tailed t-test or one way ANOVA with Tukeys post-test.
